# Nucleated red blood cells impact DNA methylation and expression analyses of cord blood hematopoietic cells

**DOI:** 10.1186/s13148-015-0129-6

**Published:** 2015-09-11

**Authors:** Olivia M. de Goede, Hamid R. Razzaghian, E. Magda Price, Meaghan J. Jones, Michael S. Kobor, Wendy P. Robinson, Pascal M. Lavoie

**Affiliations:** Child & Family Research Institute, 950 W 28th Avenue, Vancouver, BC V5Z 4H4 Canada; Department of Medical Genetics, University of British Columbia, Vancouver, BC V6T 1Z3 Canada; Department of Pediatrics, University of British Columbia, Vancouver, BC V6T 1Z3 Canada; Department of Obstetrics and Gynecology, University of British Columbia, Vancouver, BC V6T 1Z3 Canada; Centre for Molecular Medicine and Therapeutics, Child and Family Research Institute, Vancouver, BC V5Z 4H4 Canada

## Abstract

**Background:**

Genome-wide DNA methylation (DNAm) studies have proven extremely useful to understand human hematopoiesis. Due to their active DNA content, nucleated red blood cells (nRBCs) contribute to epigenetic and transcriptomic studies derived from whole cord blood. Genomic studies of cord blood hematopoietic cells isolated by fluorescence-activated cell sorting (FACS) may be significantly altered by heterotopic interactions with nRBCs during conventional cell sorting.

**Results:**

We report that cord blood T cells, and to a lesser extent monocytes and B cells, physically engage with nRBCs during FACS. These heterotopic interactions resulted in significant cross-contamination of genome-wide epigenetic and transcriptomic data. Formal exclusion of erythroid lineage-specific markers yielded DNAm profiles (measured by the Illumina 450K array) of cord blood CD4 and CD8 T lymphocytes, B lymphocytes, natural killer (NK) cells, granulocytes, monocytes, and nRBCs that were more consistent with expected hematopoietic lineage relationships. Additionally, we identified eight highly differentially methylated CpG sites in nRBCs (false detection rate <5 %, |Δ*β*| >0.50) that can be used to detect nRBC contamination of purified hematopoietic cells or to assess the impact of nRBCs on whole cord blood DNAm profiles. Several of these erythroid markers are located in or near genes involved in erythropoiesis (*ZFPM1*, *HDAC4*) or immune function (*MAP3K14*, *IFIT1B*), reinforcing a possible immune regulatory role for nRBCs in early life.

**Conclusions:**

Heterotopic interactions between erythroid cells and white blood cells can result in contaminated cell populations if not properly excluded during cell sorting. Cord blood nRBCs have a distinct DNAm profile that can significantly skew epigenetic studies. Our findings have major implications for the design and interpretation of genome-wide epigenetic and transcriptomic studies using human cord blood.

**Electronic supplementary material:**

The online version of this article (doi:10.1186/s13148-015-0129-6) contains supplementary material, which is available to authorized users.

## Background

With the increased accessibility of high throughput technologies for epigenetic and gene expression studies, genome-wide approaches have gained popularity in studies of hematopoietic cell lineage relationships [1–4]. However, although genome-wide profiling of isolated blood cells can provide a large amount of information, data interpretation is notoriously difficult in mixed cell populations [5–7]. To address this issue, studies can be performed either on homogeneous cell populations or on mixed cell samples with deconvolution algorithms applied to correct for differences in cell composition [8, 9]. One concern with the former approach in blood is that red blood cells (RBCs) have been shown to engage in functional heterotopic interactions with other hematopoietic cells [10–16]. If not formally excluded using lineage markers, these interactions could impact whole-genome studies of hematopoietic cells sorted by fluorescence-activated cell sorting (FACS), particularly in cord blood which has a notable proportion of nucleated RBCs (nRBCs) [17].

The proportion of nRBCs in cord blood varies considerably between individuals. Typically, these cells represent only a few percent of the total nucleated cell count; however, they can comprise up to 50 % of all nucleated cells in some chronic hypoxic-ischemic-related pregnancy situations [17–19]. For example, higher nRBC counts have been observed in response to prenatal exposure to infection, preeclampsia, maternal obesity, diabetes, and smoking [17–22]. nRBCs are generally resistant to lysis protocols and tend to sediment in the mononuclear cell fraction during purification by density gradient centrifugation, further complicating the isolation of pure hematopoietic cell populations [23]. Depending on their proportion, the presence of nRBCs could complicate both epigenetic and gene expression studies.

Under non-pathological conditions, DNA methylation (DNAm) shows great biological differences with tissue and cell type. Clustering of adult blood cells based on their DNAm profiles is consistent with the classical model of hematopoietic lineage relationships [6, 9, 24]. However, our initial analysis of genome-wide DNAm in cord blood cell populations isolated by FACS suggested significant cross-contamination between cell types. We observed low-incidence white blood cell (WBC) heterotopic interactions with nRBCs that were undetected by traditional singlet FACS gating, due to the small size of nRBCs. Thus, to obtain pure WBC populations, we developed and implemented a stringent sorting protocol that excludes erythroid-specific surface markers. The DNAm profiles of cell populations obtained by our stringent FACS method were used (1) to evaluate the impact of nRBC contamination on the DNAm profiles of T lymphocytes and monocytes and (2) to identify nRBC-distinct DNAm markers to detect erythroid contamination in genome-wide DNAm studies.

## Results and discussion

### Heterotopic cell interactions impact genome-wide signatures of hematopoietic cells

**Fig. 1:**
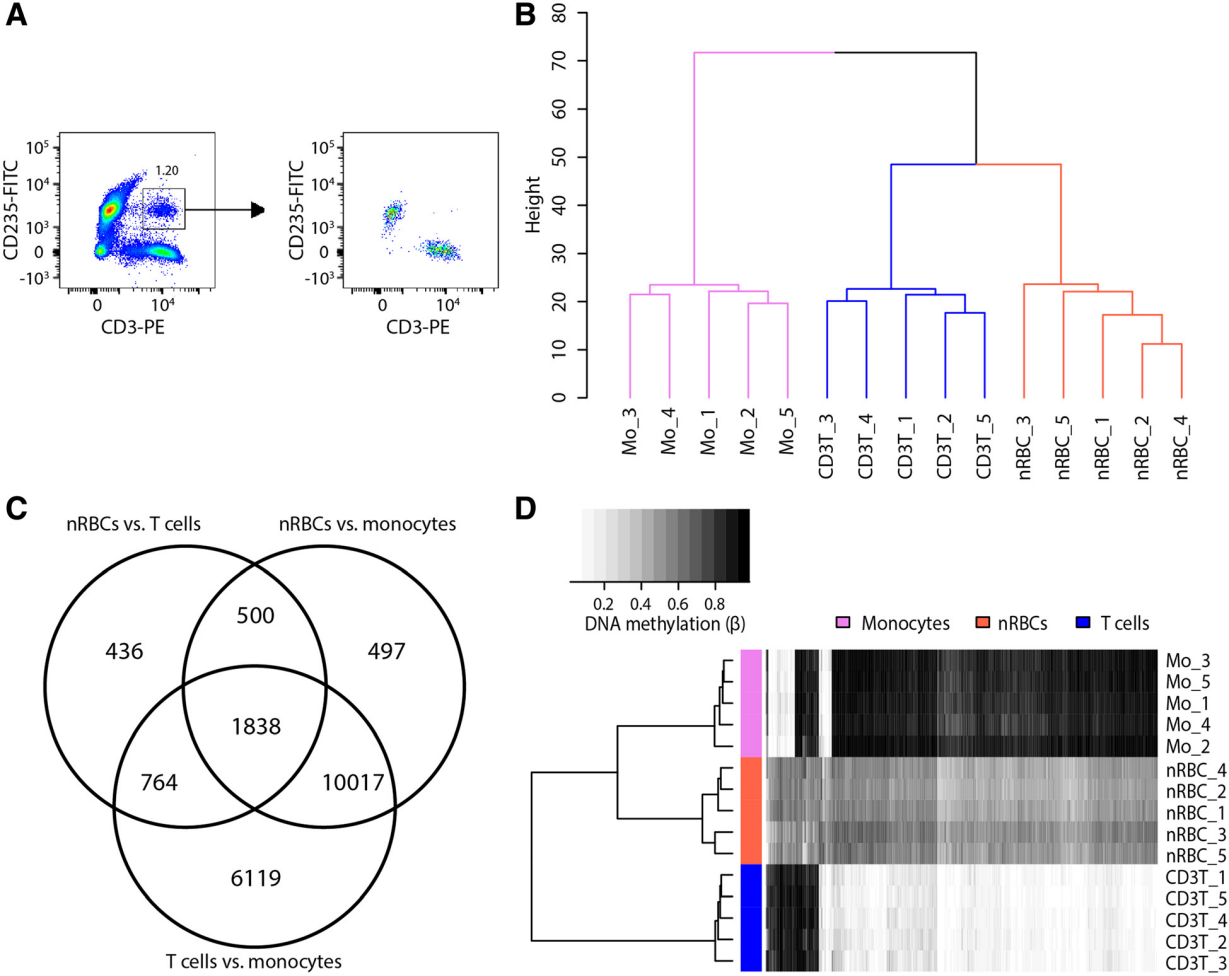
DNAm profiles of cord blood cells isolated by the standard FACS strategy. **a** A CD14-/CD19-/CD3+/CD235+ population isolated by FACS (*left panel*) is revealed to be T cell/RBC doublets by flow cytometry, which identifies two distinct cell types after sorting (*right panel*). **b** Unsupervised Euclidean clustering of genome-wide DNAm (440,315 CpG sites) between whole T cells (CD3T), nRBCs, and monocytes (Mo); *numbers* in the sample labels indicate different cord blood donors. **c** Number of large magnitude DM sites (FDR <5 %, |Δ*β*| >0.20) between nRBCs and T cells, nRBCs and monocytes, and T cells and monocytes sorted using a standard approach. **d** DNAm heatmap of nRBCs, T cells, and monocytes at top nRBC DM sites identified by the standard sorting protocol (FDR <5 %, |Δ*β*| >0.30; 457 CpG sites)

Heterotopic cell interactions are a well-known occurrence that may confound cell-specific studies. To avoid this problem, cell doublets are generally excluded during FACS by employing forward/side scatter width singlet gatings. Despite applying these standard criteria, we observed small proportions of double positive events co-expressing both T cell (CD3)- and erythroid (CD235)-specific lineage markers. When analyzed by flow cytometry after sorting, these double positive events were found to be distinct cell events expressing either erythroid or T cell markers (Fig. 1a). Examination of these sorted events under light microscopy confirmed that they were T cell-RBC doublets (not shown). To a lesser extent, we also detected events positive for expression of both erythroid (CD235) and monocyte (CD14) or B cell (CD19) markers (not shown). These findings indicate that heterotopic RBC-to-WBC doublets can be undetected by FACS using conventional singlet gating.

**Fig. 2:**
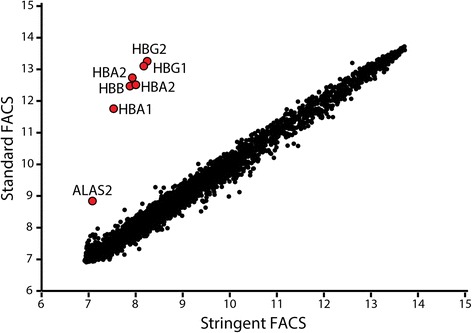
Transcriptomic profiles of naïve CD4 T cells indicate erythroid contamination when sorting by the standard, but not the stringent, FACS strategy. Log_2_(expression) of 20,876 gene probes in naïve CD4 T cells sorted by either a standard (no erythroid exclusion) or stringent (exclusion of erythroid-specific surface markers) FACS strategy. Top differentially expressed genes between these T cell populations were hemoglobin genes and *ALAS2*, a gene involved in heme synthesis, all of which exhibit increased expression in T cells sorted without formal exclusion of erythroid lineage markers

We assessed the impact of these rare heterotopic RBC-to-WBC interactions on genome-wide DNAm and gene expression analyses of hematopoietic cord blood cells. First, DNAm data from T cells, monocytes, and nRBCs sorted without formal exclusion for an erythroid cell lineage marker were evaluated. Unsupervised Euclidean clustering of array-wide DNAm showed unexpected clustering of nRBCs with T cells and monocytes as the most epigenetically distinct population (Fig. 1b). When these cell populations were compared to identify differentially methylated (DM) sites (false detection rate (FDR) <5 %, |Δ*β*| > 0.20), nRBCs versus T cells had fewer DM sites (3538) than either nRBCs versus monocytes (12,852) or T cells versus monocytes (18,738) (Fig. 1c). Even at their largest-magnitude DM sites, nRBCs sorted by the standard FACS strategy often displayed DNAm values intermediate to the DNAm of monocytes and T cells (Fig. 1d). This is unusual, since exemplar cell-specific DM sites are typically either fully methylated (*β* > 0.80) or unmethylated (*β* < 0.20), with comparison cells exhibiting opposing levels of DNAm [6, 25]. Whole-genome expression data were also compared between naïve CD4 T cells sorted by the standard FACS method and naïve CD4 T cells sorted by the stringent FACS method. We observed a high expression of hemoglobin genes in T cells sorted by the standard protocol, but not in T cells sorted by the stringent protocol (Fig. 2). Finally, to assess whether this problem also applies to other studies, we examined gene expression datasets of hematopoietic cells publicly available in the Gene Expression Omnibus. Expression of hemoglobin genes was high in the majority of cord blood hematopoietic cell datasets that we examined, indicating widespread contamination of previously published datasets (Additional file 1: Figure S2). In contrast, increased hemoglobin gene expression was not observed in hematopoietic cells collected from adult blood. Together, these findings suggest that heterotopic cell interactions, though a rare occurrence, significantly impact genome-wide molecular signatures of hematopoietic cells from cord blood.

### Revised DNAm profiles of hematopoietic cells obtained by a more stringent cell sorting strategy

**Fig. 3:**
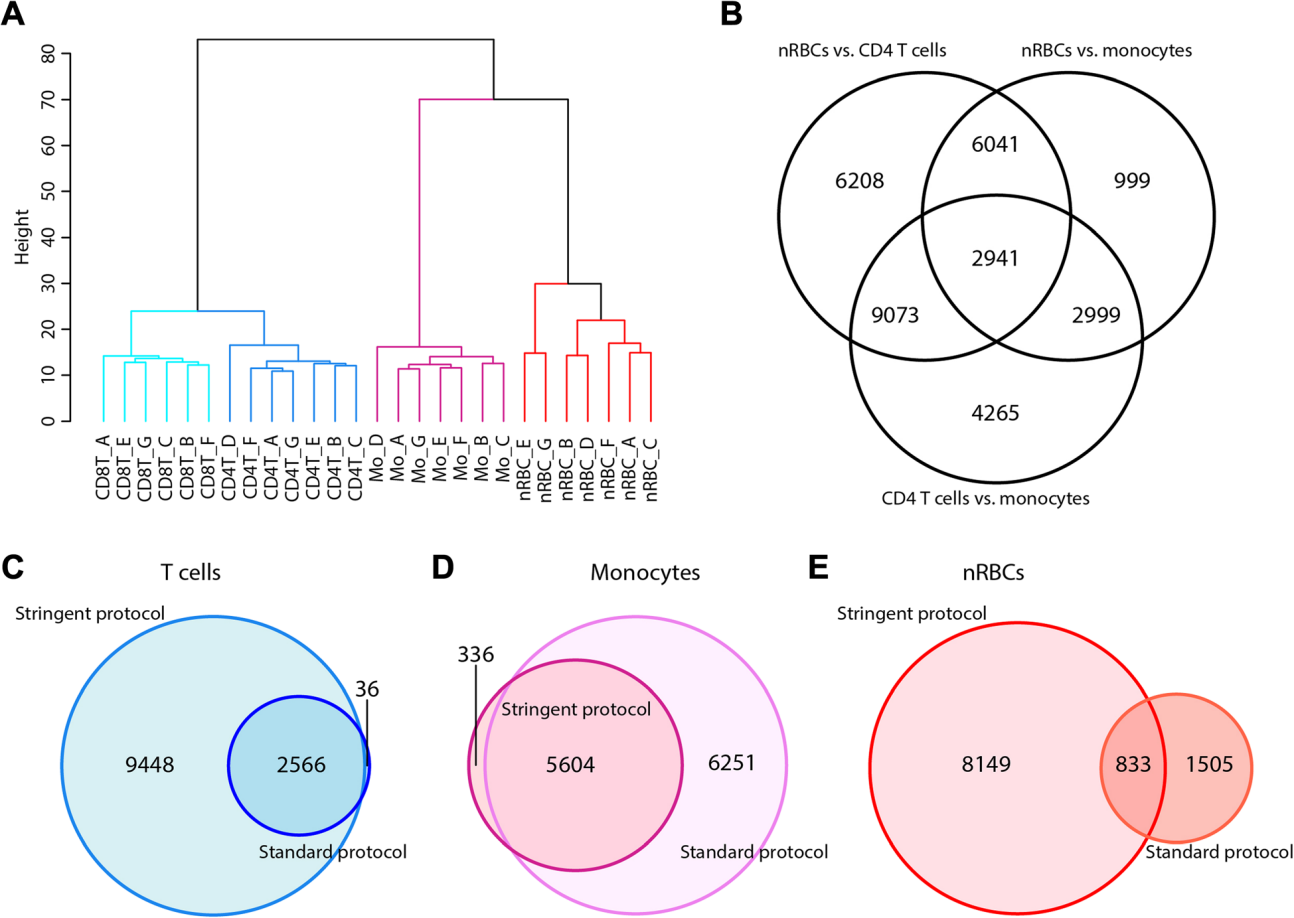
DNAm profiles of cord blood cells isolated using the stringent FACS strategy. **a** Unsupervised Euclidean clustering of genome-wide DNAm (440,315 CpG sites) in CD4 and CD8 T cells (*CD4T* and *CD8T*, respectively), monocytes (*Mo*), and nRBCs isolated by a stringent FACS protocol; *letters* in the sample labels indicate different cord blood donors. **b** Number of large magnitude DM sites (FDR <5 %, |Δ*β*| >0.20) between nRBCs and CD4 T cells, nRBCs and monocytes, and CD4 T cells and monocytes sorted using a stringent approach. **c–e** Overlap of cell-specific DM sites (FDR <5 %, |Δ*β*| >0.20) identified in the standard versus stringent sorting protocols for **c** T cells, **d** monocytes, and **e** nRBCs. Note that the stringent protocol identified a large number of additional DM sites in T cells and that the nRBC DM sites found by the two sorting protocols showed more differences than similarities

When employing a stringent sorting strategy that formally excludes RBCs, the DNAm relationships between cord blood T cells, monocytes, and nRBCs were more consistent with previous hematopoietic lineage studies [2, 26–29]. Unsupervised Euclidean clustering by array-wide DNAm showed that nRBCs were epigenetically closer to the myeloid lineage (monocytes) than to the lymphoid lineage (T cells) following this stringent sorting approach (Fig. 3a). Additionally, each hematopoietic population was more epigenetically distinct, as reflected by both principal component analysis (Additional file 1: Figure S3) and the greater number of DM sites for each cell type comparison following stringent sorting (24,263 for nRBCs versus T cells; 12,980 for nRBCs versus monocytes; 19,278 for T cells versus monocytes; Fig. 3b) compared to standard sorting (Fig. 1c). CD4 T cells and nRBCs sorted by the stringent protocol showed a greater number of cell-specific DM sites than whole (CD3+) T cells and nRBCs sorted by the standard protocol (Table 1). In contrast, monocytes sorted by the stringent protocol showed fewer DM sites, likely due to the DNAm profile of nRBCs becoming more similar to monocytes after stringent cell sorting (Fig. 3a).

**Table 1:** Number of cell-specific DM CpG sites (FDR <5 %) following the standard and stringent FACS strategies

Minimum |Δ*β*|	nRBCs, standard FACS	nRBCs, stringent FACS	Monocytes, standard FACS	Monocytes, stringent FACS	T cells, standard FACS	T cells, stringent FACS
NA	61,405	197,237	41,451	80,600	39,284	111,965
0.05	38,295	144,949	32,558	32,846	23,060	50,621
0.10	11,848	63,099	22,829	14,864	10,868	27,420
0.20	2338	8982	11,855	5940	2602	12,014
0.30	457	2628	6486	3162	879	5474
0.40	17	648	3520	1757	292	2600
0.50	0	41	1884	878	48	1268
0.60	0	1	908	319	2	553
0.70	0	0	255	51	0	158
0.80	0	0	3	1	0	12

Top DM sites for each cell type (FDR <5 %, |Δ*β*| > 0.20) were then compared between the two sorting protocols. For T cells, the majority of DM sites (>98 %) discovered by the standard method overlapped with the DM sites identified by the stringent protocol (Fig. 3c). A notable percentage (47 %) of monocyte DM sites found by the standard protocol were also discovered by the stringent protocol (Fig. 3d). For nRBCs, the DM sites identified by the two protocols showed the least overlap (36 %), with the stringent protocol identifying far more nRBC DM sites than the standard protocol (8982 versus 2338) (Fig. 3e). Of the 8982 stringent nRBC DM sites, six were located in hemoglobin genes we found to be highly expressed in cord blood WBCs sorted by a standard protocol (and thus presumed to be contaminated with RBCs) (Fig. 2; Additional file 1: Table S2). These genes were also found to be highly expressed in publicly available datasets of cord blood WBCs, again indicating widespread erythroid contamination (Additional file 1: Figure S2). The DNAm differences at these loci were striking, with the mean nRBC DNAm up to 43 percentage points less than the mean DNAm for all WBCs. Several of these CpG sites are located in either the body of the associated hemoglobin gene or within 300 bases upstream of its transcriptional start site and may be associated with erythroid-specific gene expression.

**Fig. 4:**
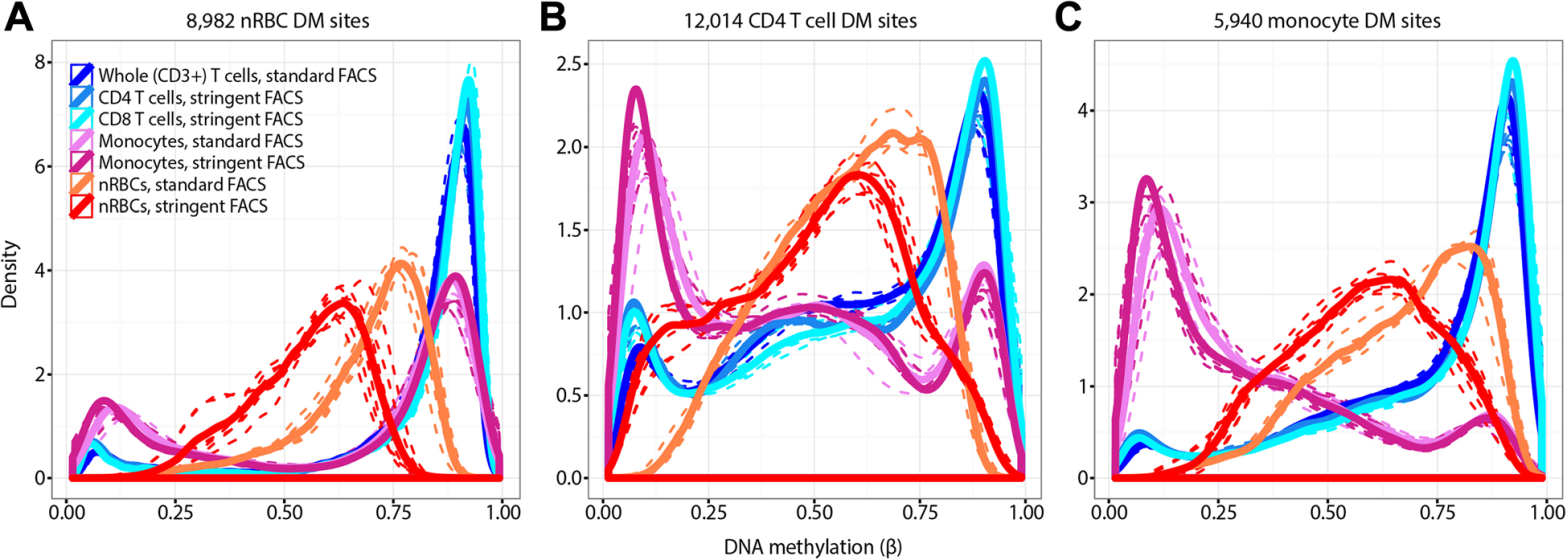
DNAm changes in nRBCs, T cells, and monocytes with FACS strategy at their top DM sites. Density distributions of DNAm (*β* values) at DM CpG sites (FDR <5 %, |Δ*β*| >0.20) for each of the three cell types, comparing the two sorting methods. DM sites were identified using data from samples sorted by the stringent protocol only. The *β* distributions suggest that nRBCs are most affected by the sorting protocol. **a** 8982 nRBC DM sites. **b** 12,014 CD4 T cell DM sites. **c** 5940 monocyte DM sites

The top DM sites from the stringent protocol represent sites with the strongest cell-specific DNAm patterns (8982 nRBC DM sites, 12,014 CD4 T cell DM sites, and 5940 monocyte DM sites). Thus, we used these sites to confirm heterotopic cell interactions in the standard protocol. The distribution of DNAm values for each cell type-by-protocol combination shows a defined shift in DNAm of the nRBC population between sorting methods (Fig. 4). When sorted by the standard method, nRBC DNAm was more similar to the DNAm patterns of T cells. The exclusion of other hematopoietic lineages in the stringent sorting of nRBCs dramatically decreased nRBC DNAm, suggesting a cleaner population of these cells. In contrast, the impact of sorting protocol on DNAm profiles of monocytes and T cells was modest.

**Fig. 5:**
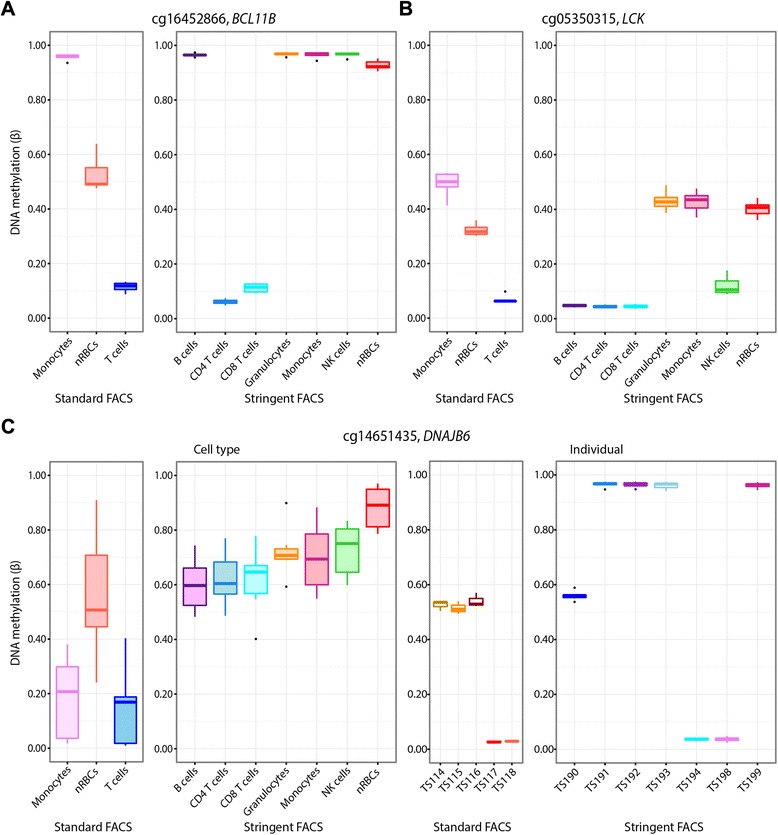
Selected discordant DM sites between the standard and stringent FACS protocols. At these discordant CpG sites, a given cell type is DM only in one protocol, but not the other. **a** An example CpG site illustrating contamination of nRBCs with T cells after sorting by standard FACS methods. The nRBCs trend toward T cell DNAm in the standard method, but are hypermethylated (like all other non-T cells) after sorting by the stringent method. **b**, **c** Examples of discordant sites in monocytes due to heterotopic cell interactions (**b**) or epipolymorphisms (**c**). In **b**, absolute DNAm in monocytes does not change significantly between the two protocols, but the change in nRBC DNAm with FACS protocol influences whether monocytes are identified as DM. In **c**, DNAm at the CpG site is likely attributable to genetic variation. This was confirmed by comparing DNAm plotted by cell type (*left boxplots*) to plotting by individual (*right boxplots*). DNAm in each individual shows one of three distinct levels of DNAm, presumably depending on the individual’s genotype at the site influencing DNAm

To further evaluate how sorting strategy affected cell type epigenetic profiles, we looked at discordant sites: sites which were DM in one sorting protocol, but not in the other. In nRBCs, differential DNAm unique to the standard protocol was observed at 1505 sites, while 8149 sites were uniquely DM in the stringent protocol (Fig. 3e). An example nRBC-discordant site is provided in Fig. 5a: a CpG site in *BCL11B* shows nRBC DNAm trending toward T cell levels in the standard FACS protocol, but exhibiting DNAm similar to other non-T cells in the stringent FACS protocol. In contrast to nRBCs, monocytes sorted by the stringent protocol had few DM sites that were not also identified in the standard protocol (Fig. 3d). Unlike nRBC-discordant sites, there appeared to be multiple reasons for monocyte-discordant sites. At some of these sites, absolute DNAm in monocytes did not change significantly between the two sorting protocols, but the change in nRBC DNAm with stringent sorting impacted the detection of differential DNAm when compared to monocytes (Fig. 5b). For other sites, DNAm differences were noted between protocols for all three cell types and may be attributable to technical noise or genetic differences between the different set of subjects for each sorting method. In fact, a few of the discordant sites were clearly “epipolymorphisms”, in which changes in DNAm levels were associated with individuals rather than cell types; this resulted in highly variable DNAm patterns within a cell type (Fig. 5c) [30].

**Fig. 6:**
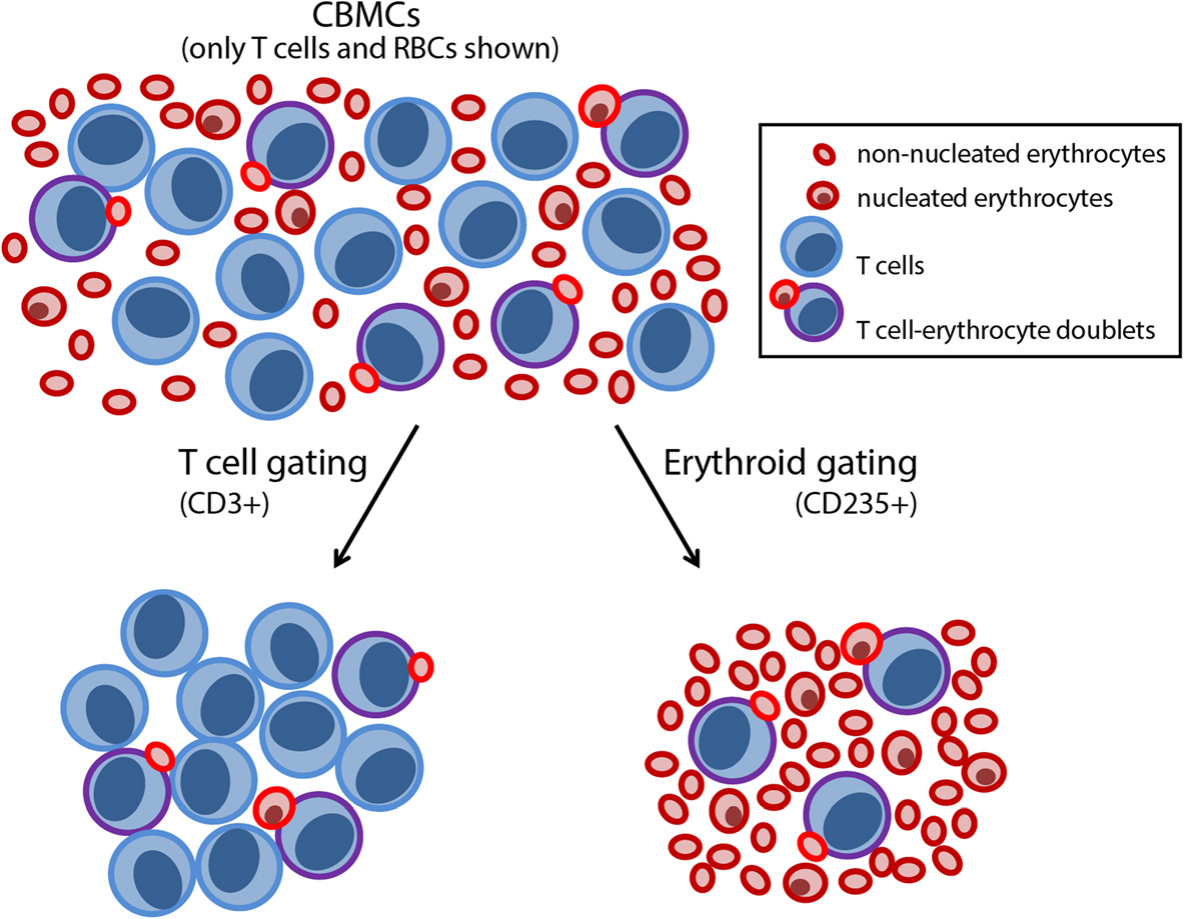
Erythroid-WBC interactions during FACS affect DNAm based on the proportion of nRBCs. As illustrated, only a fraction of erythroid cells are nucleated; therefore, contaminating nRBCs contribute less DNA than contaminating WBCs. As a consequence, heterotopic cell interactions during FACS have greater weight on the DNAm profiles of erythroid cells than of WBCs (in this figure, T cells), which are all nucleated. This illustrates how the relative impact of cross-contamination during FACS on DNAm can be much greater for nRBCs than for WBCs. However, WBC DNAm can also be strongly affected in a subset of cases where overall nRBC proportion is high

Comparing the cell-specific DM sites discovered by each sorting protocol further supports our hypothesis of heterotopic cell doublet contamination in the standard protocol. This contamination appears to have a much greater effect on nRBC DNAm than on T cell or monocyte DNAm. We attribute this to the relatively low proportion (~5–10 %) of RBCs that was nucleated in our samples (Fig. 6). The impact of heterotopic contamination by WBCs on the DNAm profile of sorted nRBCs is more obvious than the reverse, since all WBCs are nucleated. However, since the proportion of nRBCs can be as high as 50 % of all nucleated cells in cord blood [17–19], we expect that cross-contamination during FACS can have a major impact on the DNAm profile of sorted WBCs in a subset of cases.

### Erythroid-specific DM sites

To provide a way to evaluate how DNAm profiles are impacted by a mixture of blood cells, we defined erythroid lineage-specific DNAm markers. DNAm of B cells, CD4 and CD8 T cells, granulocytes, monocytes, and natural kills (NK) cells sorted using the stringent FACS strategy were compared to stringently sorted nRBCs. For each WBC population, over 210,000 of the 440,315 CpG sites analyzed showed statistically significantly different DNAm from nRBCs (FDR <5 %). Eight of these DM sites, termed erythroid DNAm markers, were selected based on a cell type average difference in DNAm >0.50 between nRBCs and every other cell type (Table 2; Additional file 1: Figure S4). We did not consider gene function in marker selection, as the intention is to use these erythroid DNAm markers to quickly detect nRBC contamination in cell samples using targeted methylation assays, such as pyrosequencing. At all eight CpG sites, nRBCs are hypomethylated compared to WBCs; thus, if a sample is significantly contaminated, DNAm should be notably lower than reference WBC levels (Additional file 1: Figure S4).

**Table 2:** Top eight CpG sites with nRBC-distinct DNAm from white blood cells in cord blood

450K array CpG identifier	CpG location: chromosome, closest gene	Location in gene	Mean nRBC β (min., max.)	Mean non-erythroid cell β (min., max.)
cg05012676	16, *ZFPM1*	Intron	0.421	0.937
			(0.298, 0.508)	(0.896, 0.963)
cg06768361	12, *TESC*	Intron; enhancer^a^	0.336	0.924
			(0.211, 0.426)	(0.825, 0.978)
cg10018933	2, *HDAC4*	Intron; enhancer^b^	0.410	0.922
			(0.346, 0.468)	(0.890, 0.953)
cg15974642	10, *IFIT1B*	TSS200	0.362	0.939
			(0.234, 0.475)	(0.904, 0.957)
cg18168751	4, *IDUA*	TSS1500; enhancer^a^	0.435	0.951
			(0.356, 0.556)	(0.924, 0.969)
cg20555305	8, *CPSF1*	Intron	0.369	0.878
			(0.277, 0.481)	(0.823, 0.917)
cg25105522	17, *MAP3K14*	Intron; enhancer^b^	0.224	0.872
			(0.126, 0.300)	(0.750, 0.962)
cg26876834	16, *SNORA64 & RPS2*	*SNORA64*: TSS1500	0.369	0.890
		*RPS2*: Intron	(0.227, 0.440)	(0.839, 0.938)

^a^Based on UCSC Genome Browser: ENCODE enhancer- and promoter-associated histone mark (H3K4Me1) in the K562 cell line

^b^Based on Illumina 450K array annotation

Some of these erythroid DNAm markers are associated with genes that have known erythropoietic function, such as *ZFPM1* and *HDAC4* (Table 2). The zinc finger protein ZFPM1 acts as a cofactor for GATA-1, a key transcription factor in erythroid differentiation [31, 32]. Histone deacetylase 4 (HDAC4) directly associates with GATA-1 and its expression is specifically reduced during erythroid maturation, likely being localized to the nucleus [33]. HDAC4 may be involved in the enucleation process of nRBCs: histone deacetylation by HDACs is essential for heterochromatin formation, and condensed chromatin is a main requirement for enucleation and terminal erythroid differentiation [34]. Interestingly, other erythroid DNAm markers are near genes involved in immune functions, such as *MAP3K14* and *IFIT1B*, consistent with the idea that nRBCs have an immunoregulatory role in early life [13]. MAP3K14 induces NF-kappa-B signaling, a major inflammatory response pathway [35]. *IFIT1* is typically silent in most cells, but becomes highly expressed in response to interferons, viral infection, and certain molecular patterns, with IFIT proteins having antiviral effects through binding and modulation of host and viral proteins and RNA [36]. As these erythroid DNAm marker sites are located largely in enhancer regions, reduced DNAm in nRBCs may reflect either specific upregulation of these genes in erythroid cells or a more primitive permissive state that is actively shut off in differentiation of other cell types.

Although these erythroid DNAm markers are the top nRBC DM sites, they display notable inter-individual variability in nRBC DNAm, with *β* value standard deviations ranging from 0.048 to 0.091. We hypothesize that this variability in DNAm may be related to important inter-individual differences in nRBC function or maturation, based on the negative Pearson correlation we observed between array-wide median nRBC DNAm and nRBC proportion (*r* = −0.86, *p* = 0.013) (Additional file 1: Figure S5A). Linear modeling identified 5935 CpG sites significantly associated (FDR <5 %) with nRBC proportion, including three of the eight CpG sites identified as erythroid DNAm markers (Additional file 1: Figure S5B–C). These results suggest that DNAm changes in cord blood nRBCs occur dynamically as a function of nRBC production and maturation, thereby revealing an additional level of functional complexity to consider in whole-genome DNAm analyses of nRBCs.

## Conclusions

While nRBCs are generally absent or rare in adult blood, they are commonly present in low proportion in cord blood, with a higher nRBC count associated with a variety of maternal and fetal health factors [17, 19–22]. Our data show that nRBCs have a distinct DNAm profile, with an association between nRBC DNAm and overall nRBC proportion in cord blood (Table 1; Figs. 3a–b and 4; Additional file 1: Figure S5). The complex DNAm profile of nRBCs has implications for epigenetic studies of whole cord blood and mononuclear cells, in which nRBCs have a demonstrable effect on DNAm (M.J.J. et al., manuscript in preparation). Despite the variability in nRBC DNAm at our identified erythroid DNAm markers, we believe that these sites will be sufficient to detect erythroid cells due to the low variation within all WBCs at these sites, as well as the large magnitude of DNAm difference between nRBCs and other cell types.

Heterotopic interactions between erythroid cells and WBCs are likely biologically meaningful events, since RBCs have immune functions that require cell-to-cell contact with WBCs. These include modulation of T lymphocyte and neutrophil survival [10, 11, 14] and immunosuppression in T and B lymphocytes and dendritic cells [12, 13, 16]. Additionally, RBCs adhere to macrophages to form erythroblastic islands during both fetal and adult RBC maturation [37]. Our data highlight the importance of formally excluding these interactions in lineage studies of cord blood hematopoietic cells using flow cytometry. This has major ramifications for the design of epigenetic, transcriptomic, and functional studies of cord blood cells.

## Availability of supporting data

The data set supporting the results of this article is available in the NCBI Gene Expression Omnibus repository, GSE68456, [http://www.ncbi.nlm.nih.gov/geo/query/acc.cgi?acc=GSE68456].

## Methods

### Ethics, consent and permissions

Ethics approval for this study was obtained from the University of British Columbia Children’s & Women’s Research Ethics Board (certificate numbers H07-02681 and H04-70488). Written, informed parental consent to participate was obtained. Individual patient data is not reported.

### Cell collection

Cord blood was collected from neonates delivered by elective cesarean section in the absence of labor at the Children’s & Women’s Health Centre of BC (Vancouver, Canada).

Hematopoietic cell purification and sorting is described in the Additional file 1: Supplemental Methods. Two sorting protocols were compared, which are referred to as the “standard” and “stringent” protocols. The stringent method includes additional negative gating steps, mainly for erythroid lineage-specific cell surface protein markers (Additional file 1: Figure S1; Additional file 1: Table S1). For DNAm studies, cells were sorted from a total of 12 subjects: whole (CD3+) T cells, monocytes, and nRBCs were collected from five individuals by the standard sorting method; B cells, CD4 T cells, CD8 T cells, granulocytes, monocytes, NK cells, and nRBCs were collected from seven individuals by the stringent sorting method. For genome-wide gene expression analysis, naïve CD4 T cells were sorted from 12 additional subjects. Transcriptomic profiling is described in the Additional file 1: Supplemental Methods.

### DNAm measurement and data normalization

DNA was extracted from isolated cell populations using standard protocols and purified with the DNeasy Blood and Tissue kit (QIAGEN, MD, USA). DNA was bisulfite-converted using the EZ DNA Methylation Kit (Zymo Research, CA, USA) before amplification and hybridization to the Illumina Infinium HumanMethylation450 BeadChip (450K array) following manufacturer’s protocols (Illumina Inc., CA, USA). With a HiScan reader (Illumina), 450 K chips were scanned.

Raw intensity data (GSE68456) were background normalized in GenomeStudio (Illumina). Quality control was performed using the 835 control probes included in the array. The intensity data were then exported from GenomeStudio and converted into M values using the lumi package [38] in R software [39]. Sample identity and quality were evaluated as described in the Additional file 1: Supplemental Methods, and one NK cell sample was removed as an outlier. The 450K array targets 485,577 DNAm sites, but probe filtering was performed as described in the Additional file 1: Supplemental Methods to produce a final dataset of 440,315 sites [40]. Red-green color bias was corrected for using the lumi package, and the data were normalized with subset within-array quantile normalization [38, 41].

### Analysis of hematopoietic cell lineage DNAm relationships

Since the stringent FACS strategy was designed based on results from the standard FACS strategy, sample collection and 450K array runs for cells collected by these two protocols were done separately. To avoid confounding by batch effects, DNAm analyses were also performed separately for the data from each FACS protocol. Our analytic approach was to compare cell types sorted by the same FACS protocol to each other and then to evaluate whether a given cell type’s epigenetic relationship with the other cell types changed between FACS methods. To eliminate DNAm differences that can arise due to genetic effects, comparisons were made between cell types derived from the same set of individuals.

For the standard sorting method, DNAm data were available for nRBCs, monocytes, and T cells from five individuals at 440,315 sites after pre-processing. Unsupervised Euclidean clustering of the samples based on DNAm *β* values was performed as an initial global analysis step. Differential DNAm between each blood cell pairing was tested by linear modeling through the R package limma [42]. Surrogate variable (SV) analysis using the R package sva [43] was performed to account for unwanted variability in the linear modeling. SVs were used as covariates in the model, with cell type as the main effect. Resulting *p* values were adjusted for multiple comparisons by the Benjamini and Hochberg [44] FDR method, and we limited statistically significant sites to those that passed an FDR <5 %. SV-corrected data was used for DNAm-based filtering of the statistically significant sites. At each site, a between-group difference in DNA methylation (Δ*β*) was calculated by subtracting mean DNAm for one cell type from the other. Differentially methylated (DM) sites were considered as those having both an FDR <5 % and |Δ*β*| >0.20.

For the stringent sorting method, DNAm data were available for B cells, CD4 T cells, CD8 T cells, granulocytes, monocytes, NK cells, and nRBCs from seven individuals at 440,315 CpG sites after pre-processing. To analyze the data in a comparable way to the standard FACS protocol, only CD4 T cells, monocytes, and nRBCs were considered. The DNAm profiles of these cell populations were analyzed as described for the standard sorting protocol.

To identify DNAm markers specific to nRBCs, data from the stringent sorting method for all seven cell types were used. DM sites between nRBCs and every other cell type were detected by linear modeling with nRBCs as the reference cell type and SVs included as covariates. Significantly, DM sites were defined as those with a FDR <5 % and a |Δ*β*| >0.50. Finally, to evaluate the relationship between nRBC proportion in whole cord blood and DNAm of nRBCs, the SV-corrected M values for the seven nRBC samples collected by stringent FACS methods were used. Linear modeling was performed with nRBC proportion (as measured by number of nRBCs/100 WBCs in whole blood) as the main effect and no covariates.

## Additional file

Additional file 1:
**Supplemental Methods and Data.** This contains Supplemental Methods, Supplemental Tables 1 and 2 and Supplemental Figures 1-5.

## Abbreviations

450K array: Illumina Infinium HumanMethylation450 BeadChip
DM: differentially methylated
DNAm: DNA methylation
FACS: fluorescence-activated cell sorting
FDR: false detection rate
NK cells: natural killer cells
nRBC: nucleated red blood cell
RBC: red blood cell
SV: surrogate variable
WBC: white blood cell
