# Supplementary material for: Nucleated red blood cells impact DNA methylation and expression analyses of cord blood hematopoietic cells
Source: Clin Epigenetics. 2015 Sep 11;7(1):95. doi: 10.1186/s13148-015-0129-6 (PMC4567832; doi:10.1186/s13148-015-0129-6)
Supplement: Additional file 1: — Supplemental Methods and Data. This contains Supplemental Methods, Supplemental Tables 1 and 2 and Supplemental Figures 1-5. [file 13148_2015_129_MOESM1_ESM.pdf]

Supplemental Methods

Cell purification

Cord blood was collected in sodium heparin anti-coagulated Vacutainers (Becton Dickinson, ON, Canada). Cord blood mononuclear cells (CBMCs) were extracted using a Lymphoprep (StemCell Technologies Inc., BC, Canada) density gradient centrifugation, washed and re-suspended in phosphate-buffered saline. B cells, CD4 and CD8 T cells, monocytes, NK cells and nRBCs were purified by FACS. The following conjugated antibodies were used to sort these cells: anti-CD3 FITC (clone HIT3a; BD Bioscience), anti-CD3 PE (clone UCHT1; BD Bioscience), anti-CD4 BV605 (clone OKT4; BioLegend, CA, USA), anti-CD8 PerCP-eFluor710 (clone SK1; eBioscience, CA, USA), anti-CD14 PE-Cy7 (clone 61D3; eBioscience), anti-CD19 Alexa Fluor 700 (clone HIB19; eBioscience), anti-CD19 PE (clone HIB19; eBioscience), anti-CD56 APC (clone MEM188; eBioscience), anti-CD71 APC (clone OKT9; eBioscience) and anti-CD235 FITC (clone 10F7MN; eBioscience). The following additional antibodies were used for cell sorting of naïve CD4 T cells for gene expression experiments: anti-CCR7 Alexa Fluor 647 (clone 3D12; BD Bioscience, ON, Canada), anti-CD25 PE-Cy7(clone M-A251; BD Bioscience) and anti-CD45RO Alexa Fluor700 (clone UCHL1; BioLegend). For the naïve T cells, samples were sorted according to the following parameters: CD3+/CD4+/CD25-/CD45RO-/CCR7+ for the standard protocol, as well as CD235- for the stringent protocol. Unstained, single-stain compensation and fluorescence-minus one (FMO) controls were prepared for each sample run. Compensation for spectral overlaps between fluorophores was done before cell acquisition. Cells were sorted on the FACSAria III (Becton Dickinson) flow cytometer using FACSDiva Software (Becton Dickinson). Data analysis was performed with Flowjo software (TreeStar, Inc., OR, USA).

Granulocytes were obtained from the bottom fraction of the Lymphoprep gradient during CBMC purification, mixed with 3% dextran/0.9% saline solution to allow separation of granulocytes from erythrocytes by sedimentation, and followed by three steps of hypotonic lysis. Hypotonic lysis was achieved by incubation of the granulocyte fraction with ice cold 0.2% sodium chloride (NaCl) for 30 seconds to lyse remaining red blood cells. Following lysis, isotonicity was restored by adding an equal volume of 1.6% NaCl solution at room temperature. Microscopic analysis using Wright-Giemsa staining indicated that the collected granulocytes were >95% neutrophils (data not shown).

Cell images were captured after Wright staining by a Nikon Eclipse E400 microscope and Canon VIXIA HFS20 camera.

RNA extraction and genome-wide expression profiling

Total RNA was extracted from the samples using QIAshredder columns and RNeasy Mini Kit (both Qiagen) according to the recommendations of the manufacturer. To ensure purity, RNA samples were cleaned using RNA Clean & Concentrator kit (Cedarlane, ON, Canada). The quantities of RNA samples were measured with a NanoDrop spectrophotometer (Thermo Fisher Scientific, DE, USA) and sample integrity was evaluated using Agilent RNA 6000 Nano kit and Agilent 2100 Bioanalyzer (Agilent, CA, USA) according to the manufacturer's instructions. All samples yielded RNA Integrity Numbers (RIN) greater than 9.3.

For whole-genome expression profiling, the RNA samples were hybridized to the Illumina HumanHT-12_v4_BeadChip array according to the recommendations of the manufacturer. The resulting data were transferred to GenomeStudio (Illumina), then further processed and

normalized in R using the lumi package.[1] Any gene probes with signal intensity <100 were considered background expression, and removed from analysis, for a final dataset of 20,876 probes. Average $\log_2$(expression) for each gene in T cells collected by the standard sorting strategy was compared to average $\log_2$(expression) for each gene in T cells collected by the stringent sorting strategy.

Quality control and probe filtering of DNAm data from 450K array

The raw intensity data produced by the 450K array were background normalized in GenomeStudio (Illumina). Quality control was performed using the 835 control probes included in the array. The intensity data were then exported from GenomeStudio and converted into M values using the lumi package[1] in R software.[2] Sample identity and quality were then evaluated in three ways: (i) clustering with the 65 SNP probes provided on the array, with samples from the same individual grouping together as expected; (ii) clustering with probes on the X and Y chromosomes, with samples grouping by known sex as expected; (iii) clustering based on all probes, producing groups based on cell type. Based on these checks, one NK cell sample was removed as an outlier. The 450K array targets 485,577 CpG sites, but probes were removed from analysis if they fell into any of the following categories: (i) probes that target SNPs (n = 65); (ii) probes that target or cross-hybridize with sites on the sex chromosomes (n = 11,648 and 11,359, respectively); and (iii) probes that target CpGs which may also contain SNPs (n = 19,271).[3] Probes that had a detection p-value>0.01 or under 3 bead replicates in more than one sample were also removed (n = 2,919), for a final dataset of 440,315 CpG sites.

References

1. Du P, Kibbe WA, Lin SM. lumi: A pipeline for processing Illumina microarray. *Bioinformatics*. 2008;24(13):1547-1548.

2. R Core Team. R: A language and environment for statistical computing. R Foundation for Statistical Computing, Vienna, Austria. 2014.

3. Price ME, Cotton AM, Lam LL, et al. Additional annotation enhances potential for biologically-relevant analysis of the Illumina Infinium HumanMethylation450 BeadChip array. *Epigenetics Chromatin*. 2013;6(1):4-8935-6-4.

Supplemental Data

Supplemental Table 1. Summary of surface antigens targeted to sort each cord blood hematopoietic cell type using the standard and stringent FACS protocols. “+”and “–” symbols respectively indicate positive and gating for a specific antigen. “•” indicates that the antigen was not used to sort a given cell type. Granulocytes were not sorted by FACS, but collected by density gradient separation, erythrocyte sedimentation, and erythrocyte hypotonic lysis.

Standard-sorted cells	CD3	CD4	CD8	CD14	CD19	CD56	CD71	CD235
Whole (CD3+) T cells	+	•	•	•	-	•	•	•
Monocytes	•	•	•	+	-	•	•	•
nRBCs	•	•	•	-	-	•	+	+
Stringent-sorted cells	CD3	CD4	CD8	CD14	CD19	CD56	CD71	CD235
B cells	-	•	•	-	+	•	•	-
CD4 T cells	+	+	-	-	-	•	•	-
CD8 T cells	+	-	+	-	-	•	•	-
Monocytes	-	•	•	+	-	•	•	-
NK cells	-	•	•	-	-	+	•	-
nRBCs	-	•	•	-	-	•	+	+
Granulocytes	*n/a*							

Supplemental Table 2. Six nRBC-DM sites (FDR <5%, |Δβ| >0.20) located in erythroid-specific (globin) genes.

450K array CpG identifier	CpG location: chromosome, closest gene	Location in gene	Mean nRBC β (min., max.)	Mean non-erythroid cell β (min., max.)
cg18764164	11, *HBB*	TSS200	0.478 (0.355, 0.582)	0.904 (0.843, 0.935)
cg14544583	11, *HBB*	TSS1500; enhancer*	0.709 (0.647, 0.773)	0.940 (0.912, 0.962)
cg18768582	11, *HBG1*	Intron	0.570 (0.466, 0.666)	0.842 (0.788, 0.884)
cg20896063	11, *HBG2*	Intron	0.371 (0.237, 0.474)	0.714 (0.662, 0.761)
cg12559170	11, *HBG2*	Intron	0.404 (0.273, 0.511)	0.658 (0.570, 0.734)
cg27009246	11, *HBG2*	TSS200	0.460 (0.241, 0.608)	0.897 (0.768, 0.954)

*Based on UCSC Genome Browser: ENCODE Enhancer- and promoter- associated histone mark (H3K4Me1) in K562 cells

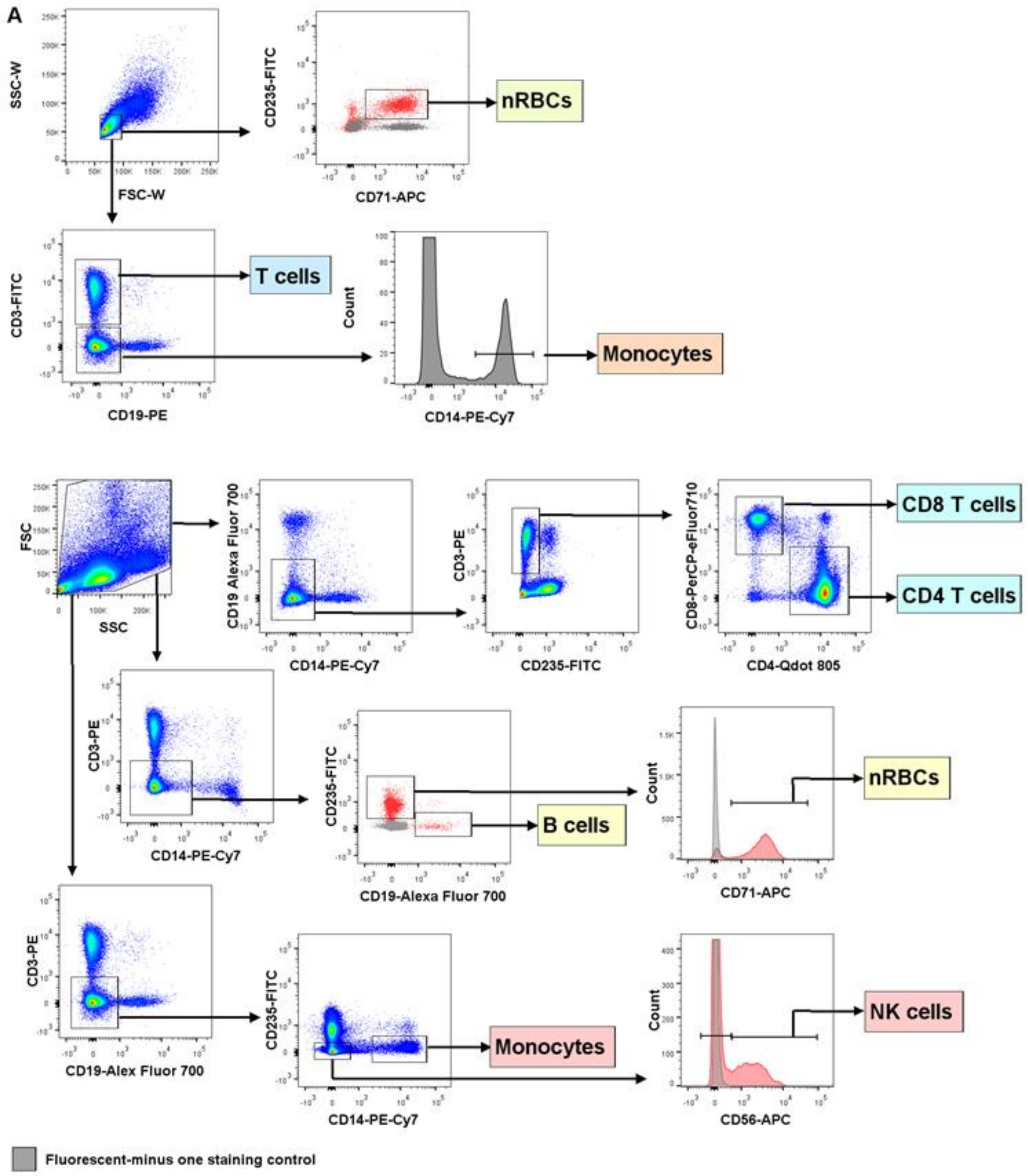

Supplemental Figure 1. Standard and stringent FACS gating strategies. Schematic representation of **(A)** the standard cell sorting strategy used to purify whole (CD3+) T cells, nRBCs, and monocytes by FACS; and **(B)** the stringent cell sorting strategy used to purify CD4 and CD8 T cells, B cells, nRBCs, monocytes, and NK cells by FACS.

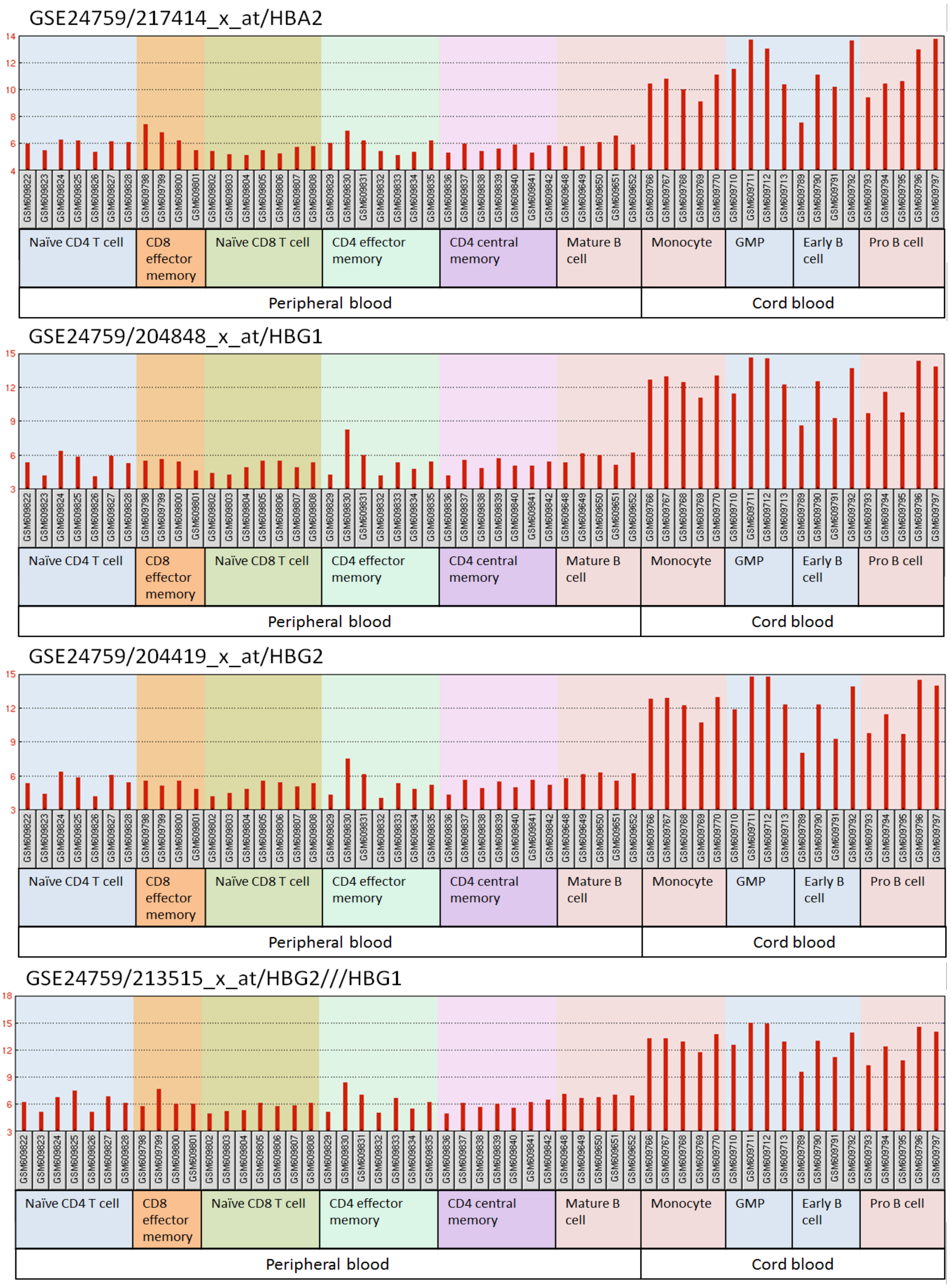

Supplemental Figure 2. Cord blood hematopoietic cells in a published Gene Expression Omnibus dataset (GSE24759) show high expression of hemoglobin genes. Expression of hemoglobin alpha, beta, and gamma genes in hematopoietic cells isolated from peripheral and cord blood by flow cytometry are reported on a log2 scale. Peripheral hematopoietic cells do not show hemoglobin gene expression above 6, the threshold for background expression. In contrast, cord blood hematopoietic cells display hemoglobin gene expression higher than background, indicating significant erythroid contamination of these isolated cell populations.

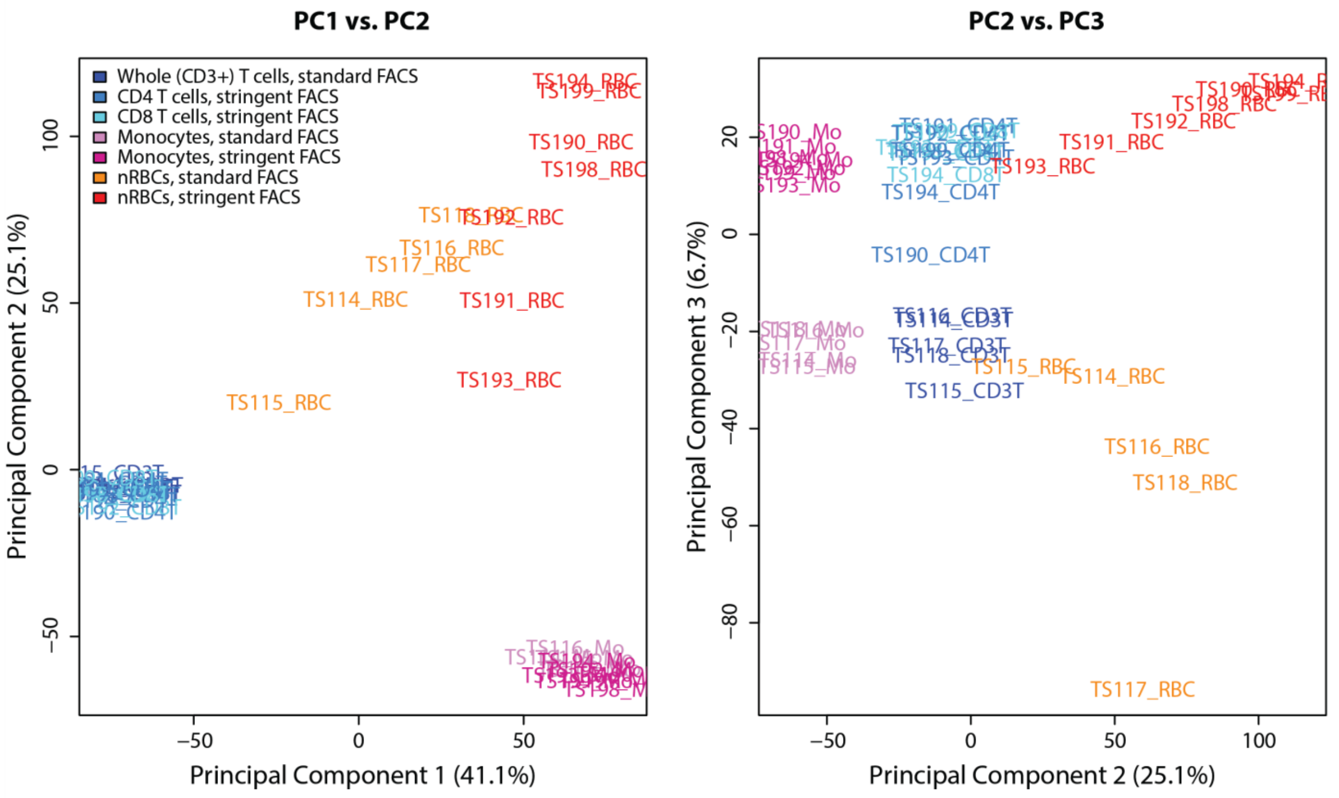

Supplemental Figure 3. Principal component analysis of T cells, monocytes, and nRBCs sorted by the standard and stringent FACS strategies. Principal components (PCs) 1 and 2 are both associated with cell type, and PC3 is associated with batch/sorting protocol. DNAm in nRBCs is strongly affected by the change in sorting protocol, but there is minimal impact on DNAm in T cells and monocytes. These PC plots are a direct comparison of cell populations from the two sorting protocols, unlike other DNAm analyses performed on these data; as such, all DNAm data were combined for SV adjustment to perform this PC analysis.

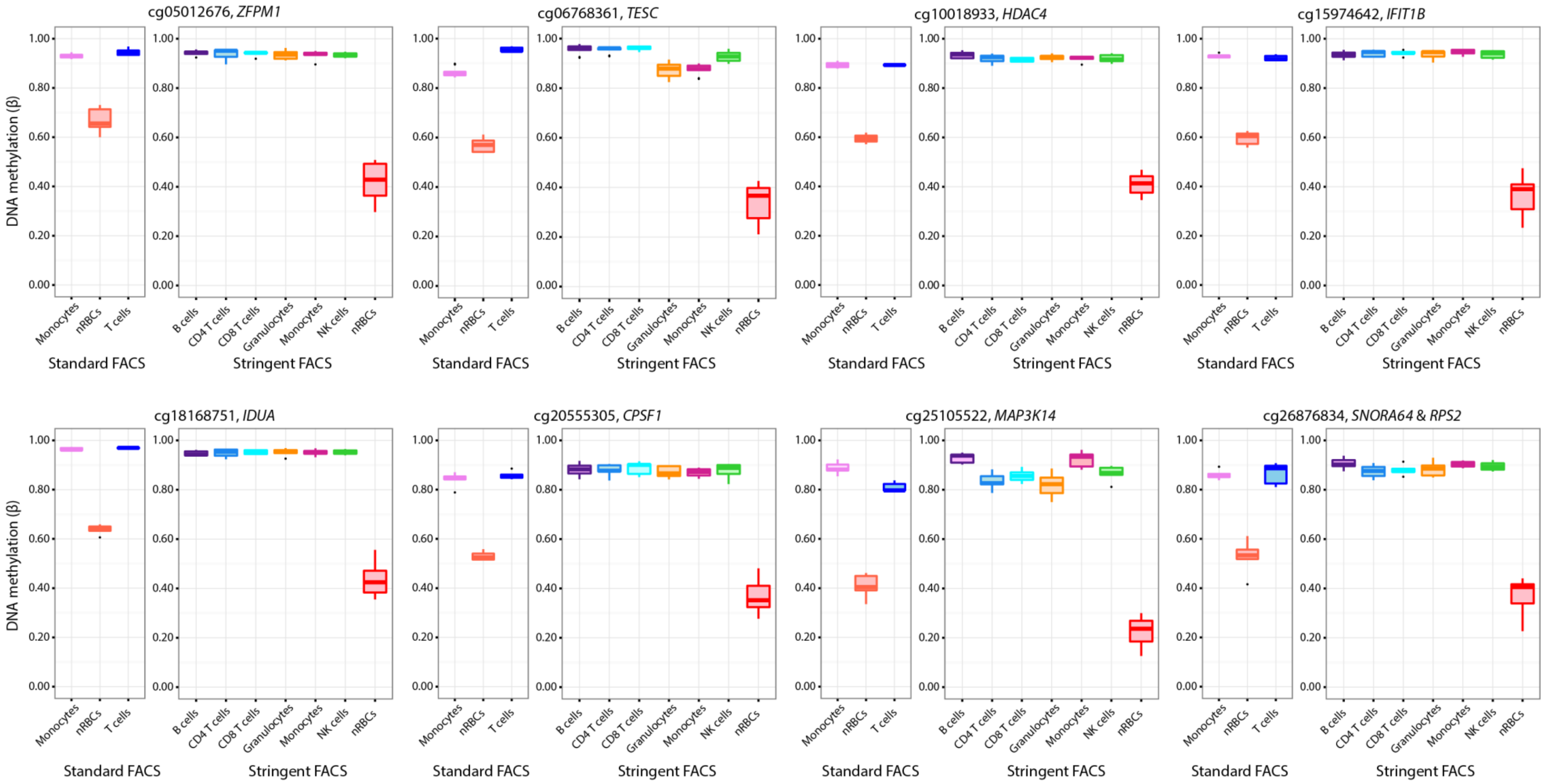

Supplemental Figure 4. DNAm in cord blood cells at eight nRBC marker sites. DNAm of B cells, CD4 T cells, CD8 T cells, granulocytes, monocytes, NK cells, and nRBCs from cord blood at the top eight CpG sites at which nRBCs are significantly DM from each of the other cell types (FDR <5%, Δβ >0.50).

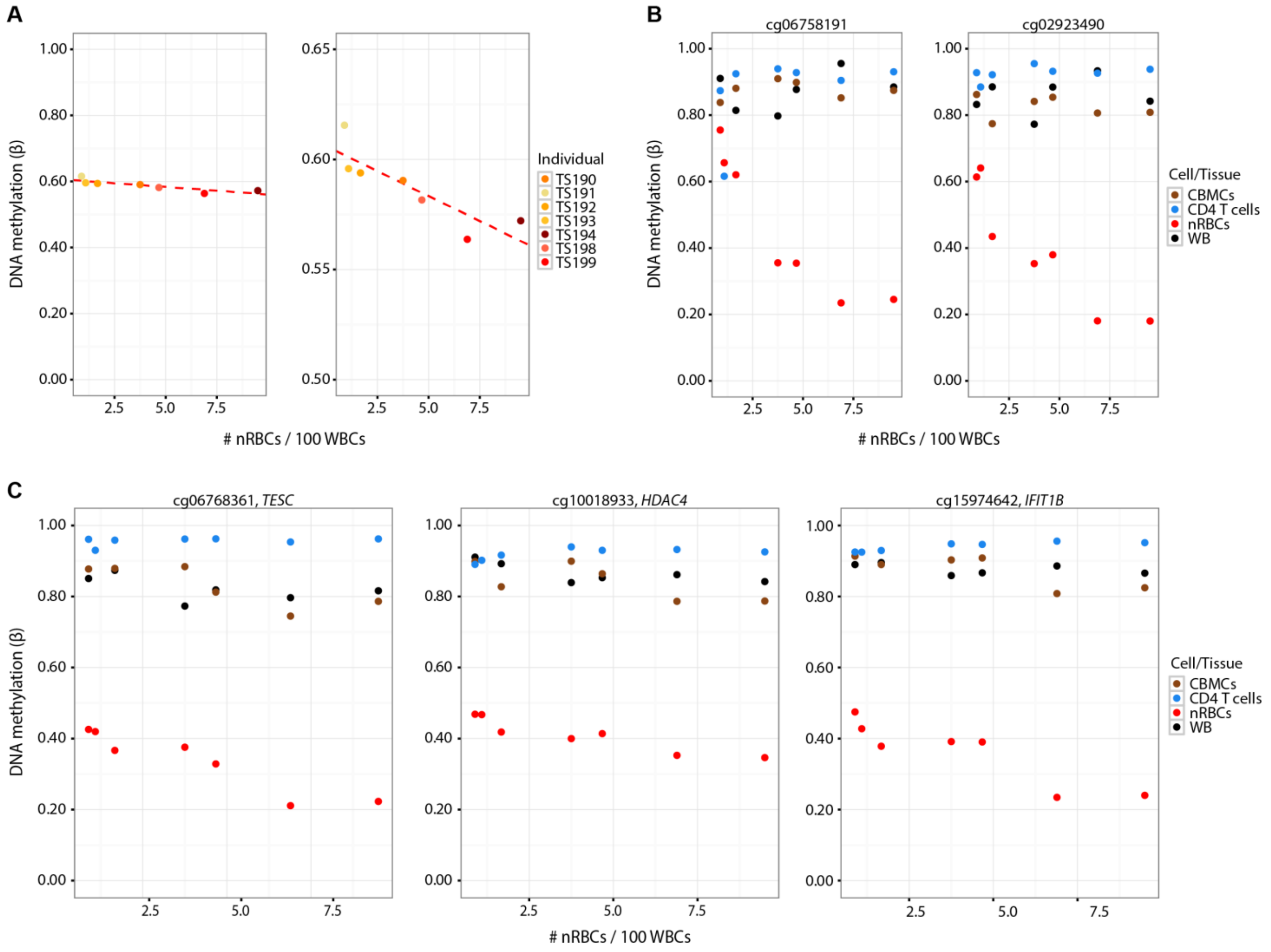

Supplemental Figure 5. DNAm in nRBCs is influenced by their proportion in whole cord blood, as measured by number of nRBCs/100 WBCs. (A) The 450K array-wide median methylation in nRBC samples differed significantly with nRBC proportion, showing a pattern of decreasing DNAm with increasing nRBC count (full y-axis scale of 0.00-1.00 on the left, narrower y-axis scale on the right for a closer look at the association). **(B)** DNAm in nRBCs, CBMCs, whole cord blood (WB), and CD4 T cells at two of the CpG sites with the strongest association between nRBC DNAm and nRBC proportion (FDR <5%, magnitude of regression coefficient > 0.05). CBMCs and whole cord blood are included to display the potential impact these DNAm changes in nRBCs could have on cord blood cell mixtures; CD4 T cells are included as a reference for the other blood cell types, which do not show an association with nRBC count. **(C)** DNA methylation in nRBCs, CBMCs, whole cord blood, and CD4 T cells at the three of our eight identified erythroid DNAm marker CpGs that are significantly associated with nRBC proportion.
